# Evaluating the forecasting performance of ensemble sub-epidemic frameworks and other time series models for the 2022–2023 mpox epidemic

**DOI:** 10.1098/rsos.240248

**Published:** 2024-07-03

**Authors:** Amanda Bleichrodt, Ruiyan Luo, Alexander Kirpich, Gerardo Chowell

**Affiliations:** ^1^ Department of Population Health Sciences, School of Public Health, Georgia State University, Atlanta, GA, USA

**Keywords:** mpox, ensemble, *n*-sub-epidemic framework, sub-epidemic wave framework, statistical models, model benchmarking

## Abstract

During the 2022–2023 unprecedented mpox epidemic, near real-time short-term forecasts of the epidemic’s trajectory were essential in intervention implementation and guiding policy. However, as case levels have significantly decreased, evaluating model performance is vital to advancing the field of epidemic forecasting. Using laboratory-confirmed mpox case data from the Centers for Disease Control and Prevention and Our World in Data teams, we generated retrospective sequential weekly forecasts for Brazil, Canada, France, Germany, Spain, the United Kingdom, the United States and at the global scale using an auto-regressive integrated moving average (ARIMA) model, generalized additive model, simple linear regression, Facebook’s Prophet model, as well as the sub-epidemic wave and *n*-sub-epidemic modelling frameworks. We assessed forecast performance using average mean squared error, mean absolute error, weighted interval scores, 95% prediction interval coverage, skill scores and Winkler scores. Overall, the *n*-sub-epidemic modelling framework outcompeted other models across most locations and forecasting horizons, with the unweighted ensemble model performing best most frequently. The *n*-sub-epidemic and spatial-wave frameworks considerably improved in average forecasting performance relative to the ARIMA model (greater than 10%) for all performance metrics. Findings further support sub-epidemic frameworks for short-term forecasting epidemics of emerging and re-emerging infectious diseases.

## 1. Introduction

In May 2022, public health officials noted an unprecedented global surge in mpox (formally monkeypox) cases in multiple countries previously free of the disease [1]. Early on, little was known regarding critical transmission and control factors—vaccination, the role of asymptomatic individuals and transmission pathways—which shaped the variable epidemic trajectories noted in the over 111 impacted countries [2–4]. Therefore, as mpox cases increased in late July 2022, our team started to produce and make publicly available weekly short-term forecasts of new cases for the highest-affected countries (e.g. Brazil, Canada, France, Germany, Spain, the United Kingdom and the United States) and at the global level [5–7]. As part of our forecasting efforts, we employed semi-mechanistic growth models [8], which have shown previous forecasting success in the context of emerging infectious diseases [7,9,10].

For any emerging pathogen that rapidly transmits throughout a population, short-term forecasts of the epidemic’s trajectory at different spatial scales can help guide policy and intervention strategies [11–16]. However, there is little opportunity to assess forecasting performance and improve models amid an ongoing public health crisis. Fortunately, as of September 2022, mpox cases have steadily declined worldwide [17–19], with non-endemic countries reporting 92 982 cases and 163 deaths overall as of 8 May 2024 [4]. Therefore, given the heterogeneous impact of the epidemic at different spatial scales and the substantial decline in mpox cases, a retrospective evaluation of the employed forecasting methodologies is vital to prepare for future public health events.

Several methodologies have been employed to forecast the trajectory of the 2022–2023 mpox epidemic in various geographical regions, including, but not limited to, models focused on human judgement [20], deep learning and artificial intelligence models [21,22], machine learning models [21–25], statistical models such as auto-regressive integrated moving average (ARIMA) models [21,23–27], compartmental models [28] and semi-mechanistic sub-epidemic models [7]. Performance metrics employed have mainly included variations of mean absolute error (MAE) [7,21,23–25,27], mean squared error (m.s.e.) [7,21–26] and mean absolute percentage error [21,23–25]. Only two studies used probabilistic measures of performance, such as the weighted interval score (WIS) [7,29]. Of the studies included here, four focused on the ascending phase of the epidemic (January–mid-August 2022) [20,24–26], three included forecasts of the epidemic’s ascension and peak (February–September 2022) [21,22,29] and four focused on the ascending, peak and declining phases of the epidemic (January 2022–present) [7,23,27,28].

In this article, we conducted a comprehensive retrospective assessment of forecasting performance (one–four weeks ahead) of semi-mechanistic sub-epidemic modelling frameworks compared against other commonly employed statistical models, including ARIMA, generalized additive models (GAM), simple linear regression (SLR) and Facebook’s Prophet model (Prophet) for the highest-impacted countries (e.g. Brazil, Canada, France, Germany, Spain, the United Kingdom and the United States) and on the global scale. We focused on producing and evaluating forecasts for the entirety of the epidemics in the countries of interest (21 July 2022 to 23 February 2023). We considered a comprehensive set of performance metrics in the field of epidemic forecasting, namely the MAE, m.s.e., 95% prediction interval (PI) coverage and WIS.

## 2. Methods

### 2.1. Data source and preparation

Our team obtained multiple daily confirmed mpox case series for the highest-impacted countries (e.g. Brazil, Canada, France, Germany, Spain, the United Kingdom and the United States) and on a global scale from the Centers for Disease Control and Prevention (CDC) [30] and the Our World in Data (OWID) GitHub page [18] on 9 and 15 August 2023, respectively. Although both data repositories employ slightly different collection and reporting methodologies, each defined a confirmed case as a laboratory-confirmed case (i.e. infection detected by polymerase chain reaction testing) [31,32]. Additional details regarding reporting methods and case obtainment for both sources can be found in electronic supplementary material, appendix S1, section A.

We aggregated cases to the weekly level to ensure an adequate number of cases for the forecasting process and to control daily reporting differences between locations. In this analysis, we defined a week as Thursday to the following Wednesday to avoid calibrating the model with partial weeks of case data. We gathered data on the global level and for the countries that experienced the most significant outbreaks during the initial epidemic wave (e.g. Brazil, Canada, France, Germany, Spain, the United Kingdom and the United States) from the OWID team [18]. Our team also retrieved United States case data from the CDC [30] to explore differences in model performance dependent on the data source used.

### 2.2. Model calibration and forecasting strategy

Our analysis included 12 models in total: the *n*-sub-epidemic framework (e.g. top-ranked, second-ranked, weighted ensemble and unweighted ensemble), the sub-epidemic wave (spatial wave) framework (e.g. top-ranked, second-ranked, weighted ensemble and unweighted ensemble), a GAM, an SLR model, ARIMA model, each with an assumption of normality and a Prophet model. Descriptions of the *n*-sub-epidemic and spatial-wave frameworks, ARIMA model, GAM and Prophet model methodologies are given below. The SLR model included time as the only predictor; therefore, its description is not included in further detail.

For each location and model, we conducted and evaluated approximately 112 retrospective weekly sequential one- to four-week forecasts, with forecasts spanning from the week of 21 July 2022 to the week of 23 February 2023. Data posted in the week of 3 August 2023 were used to produce and evaluate all forecasts. We primarily employed 11-week calibration periods for each location, with the forecast date representing the first day of the week in which the forecast was produced. However, electronic supplementary material, appendix S2 contains a sensitivity analysis comparing the forecasting performance for 9- to 11-week calibration periods (electronic supplementary material, appendix S2).

For 14 July 2022 forecast date, we could not produce forecasts for Canada for the *n*-sub-epidemic framework owing to the low case counts during the early phase of the epidemic. We also could not produce forecasts for the weeks of 14 and 21 July 2022, using the *n*-sub-epidemic and spatial-wave frameworks for Brazil owing to low case counts. Similarly, some model calibration periods were between 8 and 10 weeks long for the *n*-sub-epidemic and spatial-wave frameworks, as initial zeros (i.e. any zeros at the start of the calibration period) were truncated until the first non-zero observation occurred.

### 2.3. The *n*-sub-epidemic modelling framework

The *n*-sub-epidemic framework employs multiple epidemic trajectories modelled as the aggregation of overlapping and asynchronous sub-epidemics [8,9]. For this analysis, a single sub-epidemic followed a three-parameter generalized logistic growth model, which has displayed competitive performance in the context of varying infectious diseases, including Zika, Ebola and COVID-19 [33–36]. Further details regarding the structure of the three-parameter generalized logistic growth model can be found in electronic supplementary material, appendix S1, section B.

An *n*-sub-epidemic trajectory comprises n overlapping sub-epidemics and is given by the following system of coupled differential equations:


(2.1) $$\frac{dC_{i}\left(t\right)}{dt}=C_{i}^{\prime }\left(t\right)=A_{i}\left(t\right)r_{i}C_{i}^{p_{i}}\left(t\right)\left(1-\frac{C_{i}\left(t\right)}{K_{0_{i}}}\right).$$

The incidence curve of mpox cases is given by $\frac{dC_{i}(t)}{dt}$ , where $C_{i}(t)$ tracks the cumulative number of mpox cases for sub-epidemic i . The parameters that characterize the shape of the *i*th sub-epidemic are given by $\left(r_{i},p_{i},{K_{0}}_{i}\right)$ , for i=1,…,n . The parameter r , the growth rate per unit of time, remains positive, and parameter $K_{0}$ represents the final outbreak size. The ‘scaling of growth’ parameter p∈[0,1] allows the model to capture early sub-exponential and exponential growth patterns. If p=0 , equation (2.1) describes a constant number of new cases over time, while p=1 indicates that the early growth phase is exponential. Intermediate values of p (0<p<1) describe early sub-exponential (e.g. polynomial) growth dynamics.

Here, n represents the number of sub-epidemics considered in the epidemic’s trajectory. When n=1 , the sub-epidemic model is equivalent to the three-parameter generalized logistic growth model. However, when *n* > 1 , we employ the indicator variable, $A_{i}(t)$ , to model the onset timing of the (*i* + 1)th sub-epidemic, where (i+1)≤n . Therefore, the (*i* + 1)th sub-epidemic is triggered when the cumulative curve of the *i*th sub-epidemic exceeds the case threshold value, $C_{thr}$ (i.e. $C_{thr}\leq K_{0_{i}}$ ). Thus, we have


(2.2) $$A_{i}\left(t\right)=\left\{ \begin{array}{l} 1,\;\;\;\;\;\;C_{i-1}\left(t\right)>C_{thr}\;\;\\ 0,\;\;\;\;\;\;\;\;\;\;\;\;Otherwise\; \end{array}\right.\;\;\;\;for\;i=2,\ldots \;n,$$

where $A_{1}\left(t\right)=1$ for the first sub-epidemic. Therefore, 3n+1 parameters are needed to model a n -sub-epidemic trajectory when n>1 . This analysis considered a maximum of two sub-epidemics in the *n*-sub-epidemic trajectory ( n≤2) . The initial number of mpox cases is given by $C_{1}\left(0\right)=I_{0}$ , where $I_{0}$ is the initial number of cases in the observed data. The cumulative curve of the n -sub-epidemic trajectory is given by


(2.3) $$C_{tot}\left(t\right)\;=\sum_{i=1}^{n}C_{i}\left(t\right).$$

**Figure 1.**
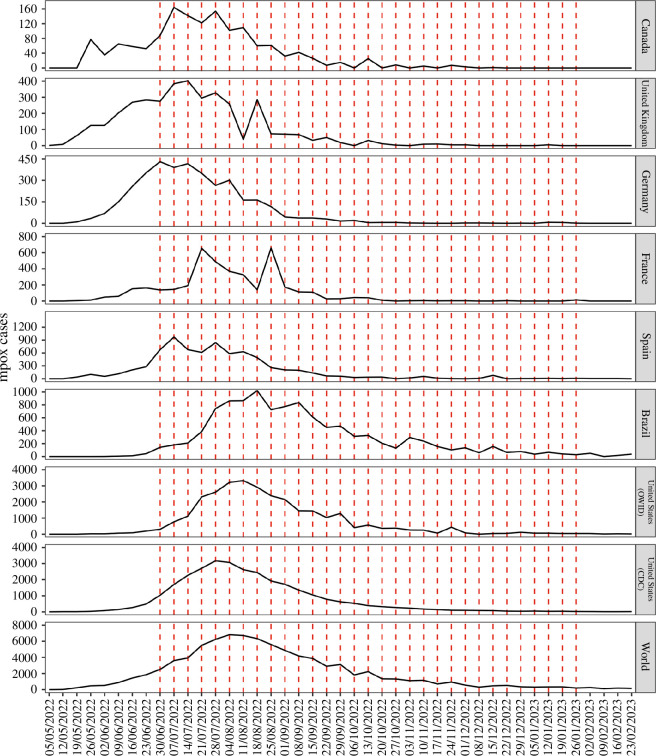
The epidemic trajectories for each study location of interest. The black solid line is the reported cases as of the week of 3 August 2023. The red-dashed lines are each of the forecasting periods included within the analysis (for all three calibration periods). Forecasts (one–four weeks) were produced through 23 February 2023, with the last forecast date being the week of 26 January 2023. Epidemic trajectories for Canada, the United Kingdom, Germany, France, Spain, Brazil, the United States (OWID) and the World are from the OWID team [18] and from the CDC for the United States (CDC) [30].

Overall, the *n*-sub-epidemic modelling framework can be applied to diverse epidemic patterns (e.g. multiple peaks and high-case level plateaus), such as the observed mpox epidemic trends (figure 1).

#### 2.3.1. Parameter estimation and model selection

We employed a nonlinear least-squares method [8] to estimate the model parameters by fitting the model solution to the observed mpox data. Subsequently, we selected the top-ranked sub-epidemic models using the corrected Akaike information criterion ( ${AIC}_{c}$ ) values of the set of best-fit models with different $C_{thr}$ values. The ${AIC}_{c}$ is given in [37,38],


(2.4) $$AIC_{c}=n_{d}log\left(SSE\right)+2m+\frac{2m\left(m+1\right)}{n_{d}-m-1},$$

where $SSE=\sum_{j=1}^{n_{d}}{\left(f\left(t_{j},\hat{\Theta }\right)-y_{t_{j}}\right)}^{2}$ , m is the number of model parameters and $n_{d}$ is the number of data points. Parameters from equation (2.4) were estimated from the nonlinear least-squares fit, which implicitly assumes normally distributed errors. Additional information regarding the parameter estimation process can be found in electronic supplementary material, appendix S1, section C.

#### 2.3.2. Parametric bootstrapping

The *n*-sub-epidemic framework quantifies parameter uncertainty for the best-fit model, $f(t,\hat{\Theta })$ , using a bootstrapping approach, which allows the computation of standard errors and related statistics without closed-form solutions [39]. Additionally, we ran the calibrated model forward in time to generate short-term forecasts (i.e. one–four weeks ahead) with quantified uncertainty, employing the same parametric bootstrapping method presented in [39] and discussed further in electronic supplementary material, appendix S1, section D. For this analysis, we used 300 bootstrap realizations to thoroughly characterize the parameter and forecasting uncertainty.

#### 2.3.3. Constructing ensemble *n*-sub-epidemic models

We generated ensemble models from both the unweighted (equally weighted top-ranking models) and weighted combination of the two highest-ranking sub-epidemic models (i.e. top- and second-ranked) as deemed by the ${AIC}_{c_{i}}$ for the i th ranked model, where $AIC_{c_{1}}\leq \cdots \leq AIC_{c_{I}}$ and i = 1,…,I . Details regarding the calculation of the weighted and unweighted ensemble models can be found in electronic supplementary material, appendix S1, section E. The PIs associated with the ensemble models were obtained using the bootstrapping approach discussed above.

### 2.4. Sub-epidemic wave framework (spatial-wave framework)

The sub-epidemic wave, or spatial-wave modelling framework, aggregates linked, overlapping, synchronous sub-epidemics (waves) to capture complex disease trajectories of differing shapes and sizes [10,40,41]. Like the *n*-sub-epidemic framework, the spatial-wave framework has shown past success in capturing diverse epidemic waves (e.g. high-level, prolonged epidemic plateaus and multi-peak trajectories), such as those seen throughout the mpox epidemic (figure 1) [10,40].

The mathematical building block for the spatial-wave model is the same three-parameter generalized logistic growth model discussed above. However, a sub-epidemic wave consists of *n-*overlapping sub-epidemics employing the following system of coupled differential equations [41]:


(2.5) $$\frac{dC_{i}\left(t\right)}{dt}=rA_{i-1}\left(t\right)C_{i}{\left(t\right)}^{p}\left(1-\frac{C_{i}\left(t\right)}{K_{i}}\right).$$

The incidence curve of mpox cases is given by $\frac{dC_{i}(t)}{dt}$ , where $C_{i}(t)$ is the cumulative number of cases for sub-epidemic i at time t and $K_{i}$ is the size of sub-epidemic i , where i=1,…,n . The indicator parameter, $A_{i}(t)$ , is the same as given in equation (2.2). Starting from an initial sub-epidemic size, $K_{0},$ the size of subsequent sub-epidemics, $K_{i},$ decline at rate q following either an exponential or power-law function as described in electronic supplementary material, appendix S1, section F. The growth rate per unit of time is represented by r , and the ‘scaling of growth’ parameter is represented by p . Unlike equation (2.1), parameters r and p do not vary across sub-epidemics, and the value of $K_{i}$ depends on the decline rate q . Hence, a total of five parameters $\left(r,p,C_{thr},{q,K}_{0}\right)$ for i=1,…,n are needed to characterize a sub-epidemic wave composed of two or more sub-epidemics ( n≥2) and four are needed when n<2 . This analysis considered a maximum of five sub-epidemics ( n≤5) in the epidemic wave trajectory.

The ${AIC}_{c}$ used in the model selection process is given in equation (2.4). However, for the spatial-wave framework, m=5 when the number of sub-epidemics is greater than one ( n>1 ) and m=4 when working with a single sub-epidemic ( n=1 ). Like the *n*-sub-epidemic framework, the ${AIC}_{c}$ for parameter estimation assumed nonlinear least-squares fit, which implicitly assumes the errors are normally distributed.

Parameter estimation and bootstrapping, construction of the ensemble models and forecasting methodologies followed the same procedures explained for the *n*-sub-epidemic modelling framework.

### 2.5. Auto-regressive integrated moving average models

ARIMA models are commonly employed in forecasting financial [42] and weather trends [43,44] and have become a standard benchmark in disease forecasting [9,21,23–26,45]. ARIMA models consist of three parts: (i) the auto-regression (AR) part involving regressing on the most recent values of the series, (ii) the moving average (MA) of error terms occurring contemporaneously and at previous times, and (iii) the integration (I) or differencing to account for the overall trend in the data and to make the time series stable. Mathematically, an ARIMA ( p,d,q ) process is given by


(2.6) $$\phi \left(B\right){\left(1-B\right)}^{d}y_{t}=c+\theta \left(B\right)\epsilon_{t},$$

where $y_{t}$ denotes the number of mpox cases at time t and B denotes the backshift operator implying $By_{t}=y_{t-1}$ and $B\left(By_{t}\right)=B^{2}y_{t}=y_{t-2},$ etc. The auto-regressive model is given by $\phi (B)=1–\phi_{1}B-\cdots -\phi_{p}B^{p}$ , where p is the order of the model. The moving average model is given by $\theta \left(B\right)=1–\theta_{1}B-\cdots -{\theta_{q}B}^{q}$ and q is the order of the moving average model. Finally, d is the degree of differencing. With this notation, $\phi \left(B\right)y_{t}=y_{t}-{\phi_{1}y}_{t-1}-\ldots -{\phi_{p}y}_{t-p}$ , $\theta \left(B\right)\epsilon_{t}=\epsilon_{t}-\theta_{1\;}\epsilon_{t-1}+\ldots +\theta_{q\;}\epsilon_{t-q}$ and ${\left(1-B\right)}^{d}$ means conducting the differencing d times. We used the auto.arima function from the R ‘forecast’ package to select orders and build the model [46], and the forecast function from the same package was used for forecasting [47]. Additional details regarding their application can be found in [48]. Any negative predicted values were truncated at zero.

### 2.6. Generalized additive models

GAMs extend generalized linear models to allow the fitting of nonlinear trends by including a sum of unknown smooth functions of some covariates while maintaining similar levels of model explainability and simplicity [49,50]. Specific to our study with time as the only covariate, assuming that $y_{t}$ follows a normal distribution, our GAM is given as follows [48,49]:


(2.7) $$y_{t}=\beta_{0}+s\left(t\right)+\epsilon_{t},$$

where s(.) is an unknown smooth function of time and $\epsilon_{t}\sim N(0,\sigma^{2})$ . We used the GAM function in the R ‘mgcv’ package to fit this model [51], where the smooth function s(.) is represented using basis functions (i.e. building blocks for creating more complex functions via linear combination). The default setting in ‘mgcv’ uses basis splines, which are piecewise polynomial functions. Specifically,


(2.8) $$s\left(t\right)=\sum_{k=1}^{k}\beta_{k}b_{k}(t),$$

where { $b_{k}(.)$ } represent the basis functions, $\left\{\beta_{k}\right\}$ are the expansion coefficients to be estimated and *k* is the number of basis functions [49]. Here, k varied depending on the length of calibration data available for a given forecasting period. A discrete penalty was imposed on the basis coefficients to control the degree of smoothness, and the model was fitted by solving a penalized least square problem. The generalized cross-validation criterion selected the smoothness tuning parameter. A more detailed description of the model fitting methodology can be found in [48,51], and the associated predict function was used for forecasting [52]. Any negative predicted values were truncated at zero.

### 2.7. Facebook’s Prophet model

Facebook’s Prophet model [53], initially designed for business-related forecasting, has recently been applied more frequently across multiple fields, including infectious disease modelling. Specifically, the model has produced both COVID-19 [45,54,55] and mpox forecasts [21]. The model’s primary assumption is that it is ‘decomposable’; therefore, y(t) , the main trend, can be decomposed into three pieces plus an error term. Thus, the model has the following form:


(2.9) $$y\left(t\right)=g\left(t\right)+s\left(t\right)+h\left(t\right)+\epsilon_{t},$$

where *g*(*t*) is a non-periodic component used for modelling the overall trend, *s*(*t*) is a periodic component used for modelling the periodic changes over time and *h*(*t*) is an irregular events component used for modelling the irregular changes (e.g. holidays or similar events). The component *ϵ_t_* is an error of the model at time *t*.

We used the default setting of the R prophet function from the ‘prophet’ package as described in Taylor [56]. The predict function was used for forecasting from the model fit [52]. Any negative predicted values were truncated to zero. A more detailed description of the model fitting methodology can be found in [48,56].

### 2.8. Model evaluation

#### 2.8.1. Performance metrics

Forecast performance was evaluated for each retrospectively generated one–four weeks ahead forecast using data downloaded the week of 3 August 2023. Descriptions of the employed metrics, MAE, m.s.e., 95% PI coverage and WIS, can be found in [8] and in electronic supplementary material, appendix S1, section G.

#### 2.8.2. Skill scores and Winkler scores

We calculated skill scores to compare the proportion of improvement over the established ARIMA model (baseline model) for average m.s.e., MAE and WIS, using the *n*-sub-epidemic and spatial-wave frameworks as comparison models. Here, average refers to the average metric over all forecasting periods for each model, location and forecasting horizon. The formula is as follows [57]:


(2.10) $$\frac{Baseline\;Model\;–\;Comparison\;Model}{Baseline\;Model}\times 100.$$

For 95% PI coverage, we first calculated Winkler scores to allow for the quantification of the proportion of improvement over the ARIMA model, using the *n*-sub-epidemic and spatial-wave frameworks as comparison frameworks. Winkler scores were calculated as follows [57]:


(2.11) $$W_{\alpha ,t}=\left\{ \begin{array}{ll} \left(u_{\alpha .t}-l_{\alpha ,t}\right)+\frac{2}{\alpha }\left(l_{\alpha ,t}-y_{t}\right),\;\;\;\;\;\;\;\;\;\; & if\;y_{t}<l_{\alpha ,t}\\ \left(u_{\alpha .t}-l_{\alpha ,t}\right),\;\;\;\;\;\;\;\;\;\;\;\;\;\;\;\;\;\;\;\;\;\;\;\;\;\;\;\;\;\;\;\;\;\;\;\;\;\;\; & if\;l_{\alpha ,t}\leq y_{t}\leq u_{\alpha ,t}\;\;\;\;\;\\ \left(u_{\alpha .t}-l_{\alpha ,t}\right)+\;\frac{2}{\alpha }\left(y_{t}-u_{\alpha ,t}\right)\;\;\;\;\;\;\;\;\; & if\;y_{t}>u_{\alpha ,t}\;\;\;\\ \;\;\; \end{array}\right.$$

where $u_{\alpha ,t}$ is the upper bound of the 95% PI interval at time t , $l_{\alpha ,t}$ is the lower bound at time *t*, $y_{t}$ is the observed mpox case incidence at t , and α=0.05 as we were working with 95% PI intervals. We then calculated the skill scores (equation (2.10)) using the average Winkler scores, where scores were averaged across all forecasting periods for each model, location and forecasting horizon.

We selected the ARIMA model for the baseline model, as it has been frequently evaluated against other forecasting methodologies in the context of mpox [21,23–26]. Therefore, its inclusion in skill score calculations provides a more in-depth quantitative evaluation of the forecasting abilities of the *n*-sub-epidemic and spatial-wave frameworks against a well-vetted methodology.

R-studio (v. 4.3.1 (Beagle Scouts)) and MATLAB (v. R2022a) were used to produce and evaluate all forecasts.

## 3. Results

### 3.1. Overall and time-period-specific forecast performance

The *n*-sub-epidemic framework outperformed the established statistical (i.e. ARIMA, GAM, SLR and Prophet) and spatial-wave framework models across most locations. Specifically, the *n*-sub-epidemic unweighted ensemble was the most successful in Brazil, the United Kingdom, the United States (OWID) and the World. The model was followed in success by the top-ranked (Brazil) and weighted ensemble (Canada and Spain) *n*-sub-epidemic models. Nevertheless, the spatial-wave top-ranked model performed best overall in Germany, the ARIMA model in France and the GAM in the United States (CDC). Model performance varied when examining different epidemic phases with the statistical models performing best most frequently during the ascending period. The ensemble sub-epidemic frameworks performed best during the remainder of the epidemic. Overall, model performance improved as case levels declined (electronic supplementary material, appendix S3, figures A–D).

### 3.2. Country-level performance

#### 3.2.1. Brazil

**Figure 2.**
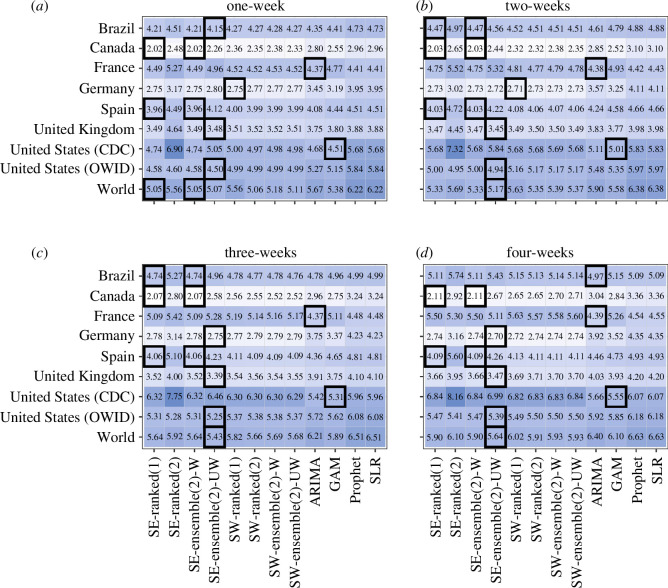
(*a*–*d*) Average m.s.e. for each location, model and forecasting horizon (weeks of 21 July 2022–23 February 2023). Approximately 1008 forecasts were produced for each model. The graph is shown on the log_10_ scale. Lighter colours indicate lower average m.s.e. scores, and darker blues indicate higher average m.s.e. scores. The black boxes indicate the best-performing model(s) for a given location and forecasting horizon. The *n*-sub-epidemic framework performed best most frequently across forecasting horizons and locations regarding average m.s.e.

**Figure 3. F3:**
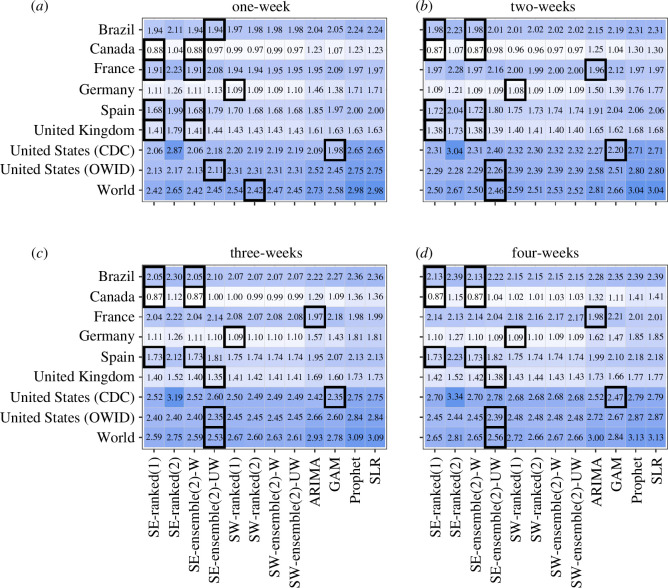
(*a*–*d*) Average MAE for each location, model and forecasting horizon (weeks 21 July 2022–23 February 2023). Approximately 1008 forecasts were produced for each model. The graph is shown on the log_10_ scale. Lighter colours indicate lower average MAE scores, and darker blues indicate higher average MAE scores. The black boxes indicate the best-performing model(s) for a given location and forecasting horizon. The *n*-sub-epidemic framework performed best most frequently across forecasting horizons and locations regarding average MAE.

**Figure 4.**
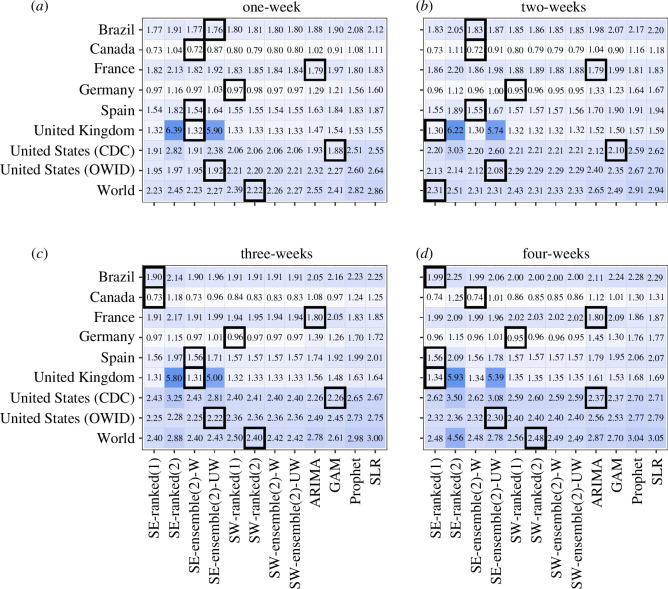
(*a*–*d*) Average WIS for each location, model and forecasting horizon (weeks of 21 July 2022–23 February 2023). Approximately 1008 forecasts were produced for each model. The graph is shown on the log_10_ scale. Lighter colours indicate lower average WIS, and darker blues indicate higher average WIS. The black boxes indicate the best-performing model(s) for a given location and forecasting horizon. The *n*-sub-epidemic framework performed best most frequently across forecasting horizons and locations regarding average WIS.

The *n*-sub-epidemic framework, specifically the top-ranked and unweighted ensemble models, performed best overall in Brazil, outperforming other models 44% of the time across performance metrics and horizons (figures 2 [Fig F3]–4–5). The *n*-sub-epidemic top-ranked and weighted ensemble models produced the lowest average m.s.e. (range: 16302.0–128915.5) and MAE (range: 86.4–135.0) across most forecasting horizons (figures 2 and 3). Both models improved between 8.5 and 29.0% over the ARIMA model regarding average m.s.e. and 21.0–33.2% for average MAE. The *n*-sub-epidemic top-ranked model most frequently produced the lowest average WIS (range: 57.4–97.5), improving 23.0–30.2% over the ARIMA model (figure 4). The *n*-sub-epidemic unweighted model produced the lowest average WIS (56.4), m.s.e. (14 240.9) and MAE (85.2) for the one-week forecasting horizon and performed best across all forecasting horizons regarding 95% PI coverage (range: 93.8–96.3%) (figures 2 [Fig F3]–4–5). When comparing average Winkler scores, the *n*-sub-epidemic unweighted ensemble improved 2.4–48.0% over the ARIMA model. Electronic supplementary material, tables A1–A5 contain the tabulation of skill scores and performance metrics across epidemic phases, models and forecasting horizons for Brazil (electronic supplementary material, appendix S4).

**Figure 5.**
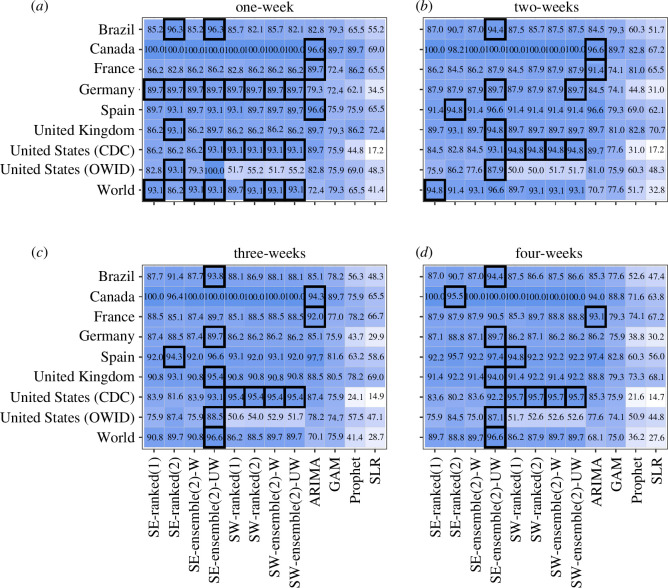
(*a*–*d*) Average 95% PI coverage for each location, model and forecasting horizon (weeks of 21 July 2022–23 February 2023). Approximately 1008 forecasts were produced for each model. Lighter colours indicate lower average 95% PI coverage, and darker blues indicate higher 95% PI coverage. The black boxes indicate the best-performing model(s) for a given location and forecasting horizon. The *n*-sub-epidemic unweighted ensemble performed best and most frequently across forecasting horizons and locations regarding average 95% PI coverage.

#### 3.2.2. Canada

The *n*-sub-epidemic weighted ensemble and top-ranked models performed best overall in Canada, outperforming other models across forecasting horizons and metrics 69 and 56% of the time, respectively (figures 2 [Fig F3]–4–5). Across all forecasting horizons for average m.s.e. (range: 103.2–128.9) and MAE (range: 6.3–6.6), the *n*-sub-epidemic top-ranked and weighted ensemble models produced the lowest values (figures 2 and 3). Both models improved considerably over the ARIMA model regarding average m.s.e. (range: 83.6–88.2%) and MAE (range: 58.8–67.6%). The success of the *n*-sub-epidemic weighted ensemble model continued for average WIS, as the model produced the lowest WIS across most forecasting horizons (range: 4.3–4.5) and improved between 54.3 and 63.0% over the ARIMA model (figure 4). However, the ARIMA model performed best and most frequently regarding average 95% PI coverage (range: 94.25–96.55%). Electronic supplementary material, tables B1–B5 contain the tabulation of skill scores and performance metrics across epidemic phases, models and forecasting horizons for Canada (electronic supplementary material, appendix S4).

#### 3.2.3. France

Unlike the other countries, the ARIMA model outperformed all other models 94% of the time across forecasting metrics and horizons, followed by the *n*-sub-epidemic top-ranked (6%) and weighted ensemble (6%) models (figures 2 [Fig F3]–4–5). The ARIMA model consistently produced the lowest average m.s.e. (range: 22986.7–24413.1), WIS (range: 61.1–62.3) and closest to 95% PI coverage on average (range: 89.7–93.1%) across forecasting horizons (figures 2, 3 and 5). Regarding average MAE, the ARIMA model performed best across most forecasting horizons (range: 90.5–93.8), albeit the one-week horizon, where the *n*-sub-epidemic top-ranked (80.9) and weighted ensemble performed best (80.9) (figure 3). However, the *n*-sub-epidemic top-ranked and weighted ensemble models saw minimal improvement over the ARIMA regarding average MAE (less than 10%). Electronic supplementary material, tables C1–C5 contain the tabulation of skill scores and performance metrics across epidemic phases, models and forecasting horizons for France (electronic supplementary material, appendix S4).

#### 3.2.4. Germany

The spatial wave top-ranked model outperformed the other models 69% of the time across forecasting horizons and performance metrics, followed by the *n*-sub-epidemic (38%) unweighted ensemble model (figures 2 [Fig F3]–4–5). The spatial-wave top-ranked model produced the lowest average m.s.e. in the one- (559.9) and two-week (514.2) forecasting horizons and across all forecasting horizons regarding average MAE (range: 11.0–11.4) and WIS (range: 7.9–8.3) (figures 2,3–4). We also noted considerable improvement in average m.s.e. (range: 80.0–86.3%), MAE (range: 59.1–72.7%) and WIS (range: 55.1–70.8%) over the ARIMA model. The *n*-sub-epidemic unweighted ensemble produced the lowest average m.s.e. during the three- (563.06) and four-week (504.64) forecasting horizons, improving 90.1–94.0% over the ARIMA model (figure 2). Similarly, the *n*-sub-epidemic unweighted ensemble performed best most frequently regarding average 95% PI coverage (89.7%), improving 30.7–65.9% over the ARIMA model in average Winkler scores (figure 5). Electronic supplementary material, tables D1–D5 contain the tabulation of skill scores and performance metrics across epidemic phases, models and forecasting horizons for Germany (electronic supplementary material, appendix S4).

#### 3.2.5. Spain

The *n*-sub-epidemic framework performed superiorly in Spain, with the most success noted for the weighted ensemble model (69%) across forecasting periods and metrics (figures 2 [Fig F3]–4–5). Regarding average m.s.e. (range: 9020.3–12286.1) and MAE (range: 46.3–52.4), the *n*-sub-epidemic top-ranked and weighted ensemble models produced the lowest values across all forecasting horizons (figures 2 and 3). Similarly, both models improved considerably over the ARIMA model regarding average m.s.e. (range: 25.7–57.4%) and MAE (range: 33.2–45.9%). The *n*-sub-epidemic weighted ensemble produced the lowest average WIS (range: 33.7–35.4) most frequently across forecasting horizons, improving 19.9–41.6% over the ARIMA model (figure 4). The *n*-sub-epidemic second-ranked model performed well regarding average 95% PI coverage, producing the closest to 95% coverage across most forecasting horizons (range: 94.3–94.8%) (figure 5). However, the ARIMA model outperformed the *n*-sub-epidemic second-ranked model across all forecasting horizons regarding Winkler scores. Electronic supplementary material, tables E1–E5 contain the tabulation of skill scores and performance metrics across epidemic phases, models and forecasting horizons for Spain (electronic supplementary material, appendix S4).

#### 3.2.6. United Kingdom

Overall, the *n*-sub-epidemic unweighted ensemble model outperformed other models 56% of the time across forecasting horizons and metrics, followed in success by the top-ranked (25%) and weighted (25%) ensemble models (figures 2 [Fig F3]–4–5). The *n*-sub-epidemic unweighted ensemble model produced the lowest average m.s.e. (range: 2454.9–3012.8) across all forecasting horizons and for the three- (21.5) and four-week (22.8) horizons regarding average MAE (figures 2 and 3). However, the model saw considerable success over the ARIMA model across all horizons in average m.s.e. (range: 45.9–72.1%) and average MAE (range: 32.1–56.6%). The *n*-sub-epidemic top-ranked and weighted ensemble produced the lowest average MAE in the one- (24.7) and two-week (22.9) horizons and saw split success across forecasting horizons regarding average WIS (figures 3 and 4). The *n*-sub-epidemic unweighted ensemble performed superiorly across most forecasting horizons for average 95% PI coverage (figure 5). Electronic supplementary material, tables F1–F5 contain the tabulation of skill scores and performance metrics across epidemic phases, models and forecasting horizons for the United Kingdom (electronic supplementary material, appendix S4).

#### 3.2.7. United States (CDC)

Unlike the other countries, the GAM model performed best in the United States (CDC), outperforming the other models 69% of the time, followed in success by the spatial-wave framework models (25%) across forecasting horizons and metrics (figures 2 [Fig F3]–4–5). The GAM produced the lowest average m.s.e. (range: 32070.5–352246.3) and MAE (range: 95.0–291.3) across all forecasting horizons and the one-week to three-week forecasting horizons for average WIS (range: 74.9–179.0) (figures 2,3–4). However, all spatial-wave framework models performed superiorly across all forecasting horizons for average 95% PI coverage (range: 93.1–95.7%), albeit the one-week horizon, where the *n*-sub-epidemic unweighted ensemble model performed equally well (93.1%) (figure 5). Nevertheless, the ARIMA model outperformed the spatial-wave framework and the *n*-sub-epidemic unweighted ensemble model across all forecasting horizons regarding average Winkler scores. Electronic supplementary material, tables G1–G5 contain the tabulation of skill scores and performance metrics across epidemic phases, models and forecasting horizons for the United States (CDC) (electronic supplementary material, appendix S4).

#### 3.2.8. United States (OWID)

The *n*-sub-epidemic unweighted ensemble model performed best across all forecasting horizons regarding average m.s.e., MAE and WIS and across most horizons regarding average 95% PI coverage, outperforming other models 94% of the time (figures 2 [Fig F3]–4–5). Regarding average m.s.e., the model produced values ranging from 31 944.2 to 246 467.4, a 66.0–82.9% improvement over the ARIMA model (figure 2). We also observed considerable improvements over the ARIMA model in average MAE (range: 50.6–60.9%) and WIS (range: 45.4–60.8%). The *n*-sub-epidemic unweighted ensemble model produced superior average 95% PI coverage across most forecasting horizons (range: 87.1–88.5%), improving 27.5–70.6% over the ARIMA model in average Winkler scores (figure 5). Electronic supplementary material, tables H1–H5 contain the tabulation of skill scores and performance metrics across epidemic phases, models and forecasting horizons for the United States (OWID) (electronic supplementary material, appendix S4).

### 3.3. World

The *n*-sub-epidemic unweighted ensemble model, followed by the spatial-wave second-ranked model, outperformed other models across forecasting horizons and metrics 56 and 31% of the time, respectively (figures 2 [Fig F3]–4–5). The *n*-sub-epidemic unweighted ensemble model produced the lowest average m.s.e. (range: 147 046.3–435 707.3) and MAE (range: 286.3–362.3) across the two- to four-week forecasting horizons, improving considerably over the ARIMA model (greater than 47%) (figures 2 and 3). Similarly, the model performed superior regarding average 95% PI coverage most frequently across forecasting horizons (range: 93.1–96.6%), albeit the two-week forecasting horizon where the *n*-sub-epidemic top-ranked model performed best (94.8%) (figure 5). The *n*-sub-epidemic unweighted ensemble model improved 42.4–56.3% over the ARIMA model regarding average Winkler scores. However, the spatial-wave second-ranked model performed best across most forecasting horizons regarding average WIS (range: 166.5–297.6), improving 53.5–59.7% over the ARIMA model (figure 4). Electronic supplementary material, tables I1–I5 contain the tabulation of skill scores and performance metrics across epidemic phases, models and forecasting horizons for the World (electronic supplementary material, appendix S4).

## 4. Discussion

In the context of the global emergency posed by the 2022–2023 mpox epidemic, we have systematically investigated the short-term forecasting performance of multiple models that have shown competitive performance during epidemics and pandemics, and rely on minimal data of the epidemic’s trajectory [7,9,10]. Overall, the *n*-sub-epidemic framework, specifically the unweighted ensemble model, followed by the spatial-wave framework, ARIMA model and GAM, performed best most frequently across locations and forecasting horizons compared with the other established modelling techniques (e.g. SLR and Prophet).

The *n*-sub-epidemic framework outperformed other models in Brazil, Canada, Spain, the United Kingdom, the United States (OWID) and the World, whereas the spatial-wave framework did best in Germany. However, France and the United States (CDC) behaved uniquely compared with other countries with the ARIMA model and GAM, respectively, performing best across forecasting horizons. Excluding France and the United States (CDC), the spatial-wave and *n*-sub-epidemic models frequently improved considerably in each metric, including in average Winkler scores, over the ARIMA model. Overall, our findings further support the sub-epidemic and ensemble frameworks for forecasting emerging infectious diseases, especially in the face of limited epidemiological data [9,10].

Although each included location experienced unique epidemic trajectories throughout the mpox epidemic, the consistent success of the *n*-sub-epidemic and spatial-wave frameworks highlights the utility of the aggregated sub-epidemic approach in capturing and producing short-term forecasts for a wide variety of epidemic trends. For example, the epidemic trajectories of the mpox epidemic in Canada, the United Kingdom and the United States (OWID) differ considerably (figure 1). Canada saw multiple peaks in cases throughout the ascending and declining phases of the epidemic. In contrast, the United Kingdom experienced a relatively smooth ascending phase with a prominent peak during the descending phase, and the United States (OWID) saw a mostly smooth epidemic trajectory without drastic peaks during the ascending or descending phases. Although different, the *n*-sub-epidemic unweighted ensemble model performed best across forecasting horizons for all three locations. Similar observations hold for Germany, Brazil, Spain and the World.

France and the United States (CDC) behaved differently from the other locations, with the ARIMA performing best in France and the GAM in the United States (CDC). The mpox epidemic in France did not follow a unimodal epidemic disease trajectory as seen in other study areas; instead, they experienced two prominent case peaks with slow ascending and descending phases (figure 1). However, throughout the outbreak, large stretches of the epidemic’s trajectory appear to form linear trends (i.e. ascending, between peaks and tail-end). As the ARIMA model inherently assumes linearity, the linear-like trajectory of mpox in France may explain the success of the ARIMA model in capturing and forecasting the disease’s trajectory [58]. Unlike France, the United States (CDC) had minimal week-to-week fluctuation, forming a smooth, unimodal epidemic trajectory (figure 1). The GAM, which performed best in the United States (CDC), captures common nonlinear trends, such as seen for the United States (CDC) epidemic trajectory [49]. Therefore, similar to France, epidemic data following distinctive trajectories may explain the success seen by the ARIMA and GAM models in France and the United States (CDC).

The model performance also differed when looking at specific epidemic phases (e.g. ascending, peak, descending and tail-end of the epidemic). For example, across locations, the established statistical models outperformed other models most frequently during the ascending phase of the epidemic. However, there were multiple epidemic phases with few forecasting periods available, limiting the ability to examine country-specific epidemic phase performance in greater detail.

Finally, the significant success of the *n*-sub-epidemic unweighted and weighted ensemble models highlights the continued utility of ensemble modelling in short-term forecasting [59–66]. Ensemble modelling has also shown considerable success against individual top-ranking sub-epidemic and other statistical models in past mpox short-term forecasting efforts [7], along with that of COVID-19 [9,61,67], seasonal influenza [62,64] and Zika [68]. Each ensemble model (e.g. weighted and unweighted) included within the analysis performed competitively at various forecasting horizons and for different areas. However, the unweighted ensemble *n*-sub-epidemic model outperformed all other included models for most locations, demonstrating the continued competitiveness of ensemble modelling in short-term mpox forecasting.

Our study is not exempt from limitations. We used two sources for weekly time-series laboratory-confirmed mpox cases, each using varying approaches to compile and disseminate case information. Similarly, the reporting patterns for countries varied as well. Therefore, the trends noted above may be a function of reporting delays rather than true case fluctuations. However, to account for the effect of reporting differences on the observed epidemic trajectories, we aggregated cases weekly to adjust for within-week reporting delays. Owing to the unprecedented nature of the epidemic and the limited epidemic data available, we examined forecasts derived from the second-ranked spatial wave and *n*-sub-epidemic models. Frequently, there was little statistical support for the second-ranked models relative to the top-ranked model for both frameworks. The spatial-wave and ensemble *n*-sub-epidemic frameworks are semi-mechanistic and provide insight into the natural processes that formed the observed epidemic trends from the aggregated sub-epidemics. Nevertheless, both frameworks do not account for explicit mechanisms of reactive behavioural modification and interventions, which probably played a significant role in controlling the epidemic at local and global levels [13,69]. Finally, current models are not responsive to seasonal or event-specific risk behaviours, which limits their application to short-term forecasts.

## 5. Conclusion

In conclusion, our findings further support the competitive performance of the aggregated sub-epidemic methodologies in producing short-term forecasts within the context of mpox, along with the utility of ensemble modelling therein producing short-term forecasts. However, the frameworks could be expanded to other disease types as they have shown utility in short-term forecasting for epidemiologically different diseases (e.g. COVID-19 and Ebola). Finally, short-term forecasting tools requiring minimal data inputs are essential in the face of unprecedented pandemics and epidemics. Therefore, further evaluation and refinements of the ensemble sub-epidemic frameworks could be achieved via comparison with other models since all the forecasting results are publicly available to the community.

## Data Availability

Data and relevant code for this research work are stored in GitHub [70] and have been archived within the Zenodo repository [71]. Supplementary material is available online [72].
